# Risk Factors and Causes of Ischemic Stroke in 1322 Young Adults

**DOI:** 10.1161/STROKEAHA.122.040524

**Published:** 2022-12-13

**Authors:** Merel S. Ekker, Jamie I. Verhoeven, Mijntje M.I. Schellekens, Esther M. Boot, Mayte E. van Alebeek, Paul J.A.M. Brouwers, Renate M. Arntz, Gert W. van Dijk, Rob A.R. Gons, Inge W.M. van Uden, Tom den Heijer, Paul L.M. de Kort, Karlijn F. de Laat, Anouk G.W. van Norden, Sarah E. Vermeer, Marian S.G. van Zagten, Robert J. van Oostenbrugge, Marieke J.H. Wermer, Paul J. Nederkoorn, Thomas P. Zonneveld, Henk Kerkhoff, Fergus A. Rooyer, Frank G. van Rooij, Ido R. van den Wijngaard, Catharina J.M. Klijn, Anil M. Tuladhar, Frank-Erik de Leeuw

**Affiliations:** 1 Department of Neurology, Radboud University Medical Centre, Donders Institute for Brain, Cognition and Behaviour, Nijmegen, the Netherlands (M.S.E., J.I.V., M.M.I.S., E.M.B., C.J.M.K., A.M.T., F.-E.d.L.).; 2 Department of Neurology, Amphia Hospital, Breda, the Netherlands (M.E.v.A., A.G.W.v.N).; 3 Department of Neurology, Medisch Spectrum Twente, Enschede, the Netherlands (P.J.A.M.B., R.M.A.).; 4 Department of Neurology, Canisius-Wilhelmina Hospital, Nijmegen, the Netherlands (G.W.v.D.).; 5 Department of Neurology, Catharina Hospital, Eindhoven, the Netherlands (R.A.R.G., I.W.M.v.U).; 6 Department of Neurology, Franciscus Gasthuis & Vlietland, Rotterdam, the Netherlands (T.d.H.).; 7 Department of Neurology, Elisabeth-TweeSteden Hospital, Tilburg (P.L.M.d.K.).; 8 Department of Neurology, Haga Hospital, The Hague, Netherlands (K.F.d.L.).; 9 Department of Neurology, Rijnstate Hospital, Arnhem, the Netherlands (S.E.V.).; 10 Department of Neurology, Jeroen Bosch Hospital, ‘s-Hertogenbosch, the Netherlands (M.S.G.v.Z.).; 11 Department of Neurology, Maastricht University Medical Centre, Maastricht, the Netherlands (R.J.v.O.).; 12 Department of Neurology, Leiden University Medical Centre, Leiden, the Netherlands (M.J.H.W.).; 13 Department of Neurology, Amsterdam University Medical Centres, Amsterdam, the Netherlands (P.J.N., T.P.Z.).; 14 Department of Neurology, Albert Schweitzer Hospital, Dordrecht, the Netherlands (Henk Kerkhoff, MD, PhD).; 15 Department of Neurology, Zuyderland Hospital, Sittard-Geleen, the Netherlands (F.A.R.).; 16 Department of Neurology, Medical Centre Leeuwarden, Leeuwarden, the Netherlands (F.G.v.R.).; 17 Department of Neurology, Haaglanden Medical Center, the Hague, the Netherlands (I.R.v.d.W.).

**Keywords:** atherosclerosis, ischemic stroke, Netherlands, prognosis, risk factors

## Abstract

Identification of risk factors and causes of stroke is key to optimize treatment and prevent recurrence. Up to one-third of young patients with stroke have a cryptogenic stroke according to current classification systems (Trial of ORG 10172 in Acute Stroke Treatment [TOAST] and atherosclerosis, small vessel disease, cardiac pathology, other causes, dissection [ASCOD]). The aim was to identify risk factors and leads for (new) causes of cryptogenic ischemic stroke in young adults, using the pediatric classification system from the IPSS study (International Pediatric Stroke Study).

**Methods:**

This is a multicenter prospective cohort study conducted in 17 hospitals in the Netherlands, consisting of 1322 patients aged 18 to 49 years with first-ever, imaging confirmed, ischemic stroke between 2013 and 2021. The main outcome was distribution of risk factors according to IPSS classification in patients with cryptogenic and noncryptogenic stroke according to the TOAST and ASCOD classification.

**Results:**

The median age was 44.2 years, and 697 (52.7%) were men. Of these 1322 patients, 333 (25.2%) had a cryptogenic stroke according to the TOAST classification. Additional classification using the ASCOD criteria reduced the number patients with cryptogenic stroke from 333 to 260 (19.7%). When risk factors according to the IPSS were taken into account, the number of patients with no potential cause or risk factor for stroke reduced to 10 (0.8%).

**Conclusions:**

Among young adults aged 18 to 49 years with a cryptogenic ischemic stroke according to the TOAST classification, risk factors for stroke are highly prevalent. Using a pediatric classification system provides new leads for the possible causes in cryptogenic stroke, and could potentially lead to more tailored treatment for young individuals with stroke.

An estimated 10% to 15% of all strokes occur in young adults (18–49 years), resulting in about 2 million young adults who are affected by stroke worldwide every year, with incidence increasing over the past decade.^1–3^ Stroke at young age comes with high socioeconomic costs and patients encounter lifelong consequences.^2,4^ Rapid identification of causes and risk factors of ischemic stroke in young adults is key to optimize treatment and prevent recurrence. Still, in up to one-third of all cases with ischemic stroke at young age, no clear cause is identified after thorough clinical work-up and the use of stroke classification schemes such as the TOAST (Trial of ORG 10172 in Acute Stroke Treatment) classification and the ASCOD (atherosclerosis, small vessel disease, cardiac pathology, other causes, dissection) classification.^5–8^

However, these classifications have been developed for ischemic stroke patients often older than 65 years. They have gradually been implemented in clinical practice of patients with stroke at younger ages, without any formal evaluation and validation in this specific domain. This may result in unjustified classification of patients with a cryptogenic stroke, while in fact causes or risk factors for stroke are present that are *not* recognized as such within the conventional developed classification schemes. In addition, the currently used classifications lump patients with diverse and rare causes and underlying pathophysiological mechanisms into 1 other determined category, thereby ignoring the possible different long-term prognosis of stroke depending by the different causes.

In contrast to the classification schemes used in adults, the classification developed for childhood- and adolescent stroke by the IPSS study (International Pediatric Stroke Study) designates not 1 single cause of stroke. The IPSS allows multiple risk factor categories that are not mutually exclusive and recognizes age-specific presumed risk factors and etiologies that are not included in the TOAST and ASCOD.^9^ Although a risk factor is not necessarily synonymous with a cause, rapid identification of risk factors is an important step to initiate treatment and counseling of patients.

We therefore investigated the causes and risk factors of stroke in young adults in a prospective cohort study of young patients with imaging proven ischemic stroke according to the TOAST, ASCOD, and IPSS criteria to search for clues that help find potential causes in cryptogenic stroke.

## Methods

### Data Availability

The raw and anonymized data used in this study can be shared upon and after permission of our institutional review board. Written proposals can be addressed to the corresponding author and will be assessed by ODYSSEY (Observational Dutch Young Symptomatic StrokE studY) investigators for appropriateness of use, and a data sharing agreement in accordance with Dutch regulations will be put in place before data are shared.

### Study Description

This study is part of the ODYSSEY, a Dutch multicenter prospective cohort study in 17 centers on the risk factors and prognosis of patients with a first-ever, ischemic stroke or intracerebral hemorrhage aged 18–49 years. Details of the study have been described previously.^10^ For this study, we included patients with ischemic stroke. Patients were included consecutively between May 2013 until end of inclusion in February 2021.

In short, our study comprises consecutive patients aged 18–49 years with first-ever symptomatic ischemic stroke with radiological evidence of cerebral ischemia. Patients with transient symptoms (<24 hours) all had diffusion weighted imaging positive lesions (DWI+) on magnetic resonance imaging and as such were included as (minor) stroke according to the tissue based definition.^11^

### Baseline Data Collection

Information on (vascular) risk factors, stroke severity according to the National Institutes of Health Stroke Scale (NIHSS) on admission and modified Rankin Scale (mRS) at discharge, treatment in the acute phase and at discharge were systematically collected as were results of neuroimaging, laboratory and cardiac diagnostic tests. Case-fatality was defined as death within the first 30 days after stroke. Case fatality was ascertained by checking all medical files, in combination with an interview by phone as part of the regular follow-up. When patients were lost to follow-up case fatality was ascertained by contacting their general practitioners. In addition, patients completed a standardized structured questionnaire on level of education, marital status, employment and additional potential risk factors such as illicit drug use. We provide a complete case analysis.

### Risk Factors and Causes of Stroke

To limit missing data, patient’s medical files, including diagnostic tests, risk factors, and the cause of ischemic stroke, were systematically assessed for all patients by 1 of 4 raters (ME, JV, MS, or EB) according to the modified TOAST criteria (including a subdivision into high-risk and medium-risk sources of cardio-embolism and in large artery atherosclerosis and likely atherothrombotic disease)^12–14^ (further referred to as TOAST), ASCOD criteria,^8^ and modified IPSS criteria (referred to as IPSS^9^; Table S1).

In case of doubt or disagreement of a risk factor of cause, a consensus meeting was held with a vascular neurologist (FEdL). The adjudicated causes and risk factors for stroke according to the TOAST, ASCOD, and IPSS classification were reviewed by 2 separate raters in the first 100 patients with an interrater agreement of 98%.

### Statistical Analyses

We reported categorical variables as numbers and frequencies unless otherwise stated. Continuous variables were reported as median and interquartile range (IQR). Age- and sex-specific analyses for causes and risk factors were performed by predefined age-groups: 18–25, 26–30, 31–35, 36–40, 41–45, and 46–49 years.

Categorical variables were compared through Pearson χ^2^ or Fisher Exact tests, whichever was appropriate. Continuous variables were compared by Student *t* test or Mann Whitney *U* test and the Kruskal-Wallis test was used for comparing median age, NIHSS, and mRS between men and women and between stroke subtypes.

For all analyses, 2-sided *P* values of <0.05 were considered statistically significant. Data were analyzed using SPSS Software version 22 (IBM), R version 3.6.2 (R Project for Statistical Computing) and Microsoft Office Excel 2007.

### Ethical Approval

The Medical Review Ethics Committee region Arnhem-Nijmegen approved the study (NL41531.091.12). Informed consent was obtained for all patients. Additional approval was obtained for Inclusion of patients that died within the first 30 days after stroke without informed consent for those who had been unable to provide informed consent.

## Results

### Demographics

In total, 1223 (92.5%) patients had an ischemic stroke with symptoms lasting longer than 24 hours, and 99 (7.5%) with symptoms <24 hours. Median age was 44.2 years (IQR [38.4–47.5]), 697 were men (52.7%), and 38 (2.9%) patients died within the first 30 days (case fatality; Table 1). Work-up was according to clinical practice and local protocols for young patients with stroke, including neuroimaging for all patients: 1114 (84.3%) patients underwent computed tomography‚ 1160 (87.7%) underwent magnetic resonance imaging‚ 953 (72.1%) both computed tomography and magnetic resonance imaging‚ 96.0% had imaging of the carotid arteries (658 [49.8%] computed tomography angiography‚ 627 [47.4%] magnetic resonance angiography‚ 517 [39.1%] an ultrasound of the carotids). An electrocardiogram was made in 1279 patients (96.7%), 1131 patients had a least 24 hours of cardiac rhythm monitoring (85.5%), 1071 patients (81.0%) had a transthoracic echocardiography, and 249 patients (18.8%) underwent a transesophageal echocardiography. Of the 213 patients (16.1%) that did not receive any cardiac ultrasound, 31 patients (2.3%) had no clear cause for their stroke. The other 182 patients (13.8%) without cardiac ultrasound had a clear other cause of stroke, deeming cardiac ultrasound unnecessary for the etiologic diagnosis. Laboratory diagnostics for antiphospholipid syndrome^15^ was performed in >70% of all patients.

**Table 1.**
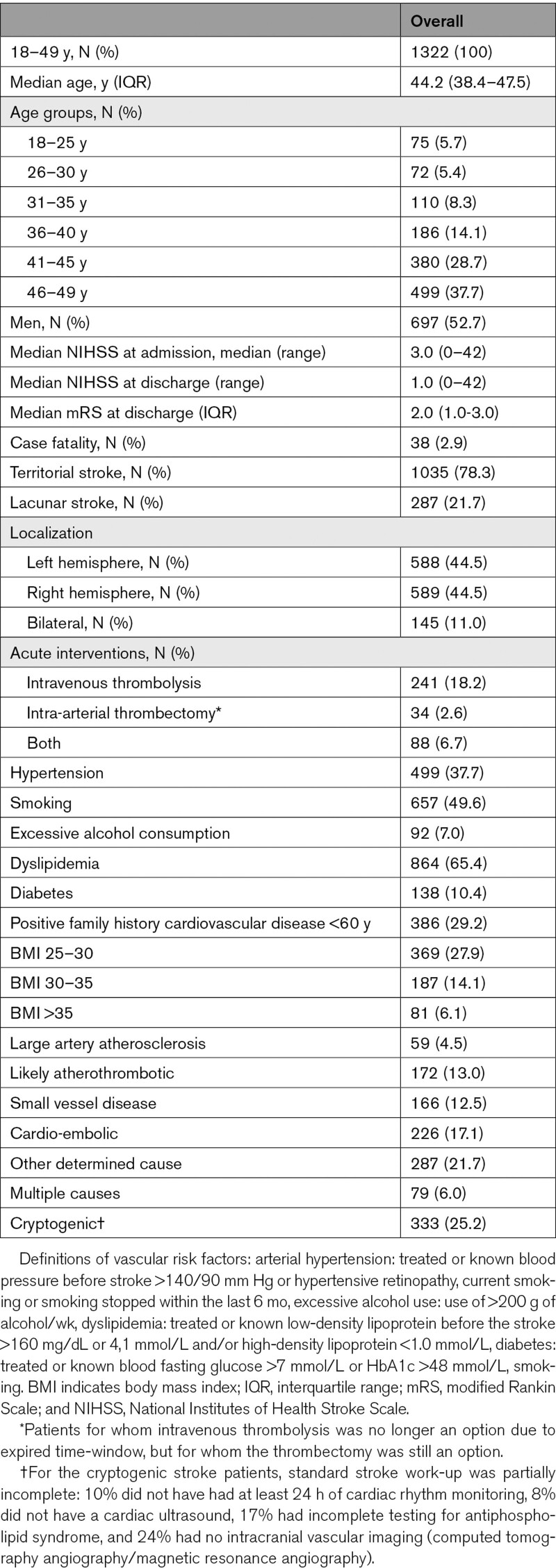
Baseline Characteristics

### Causes According to Modified TOAST Criteria, Stratified by Age and Sex

Large artery atherosclerosis was identified as the cause of stroke in 59 (4.5%) patients, likely atherothrombotic stroke in 172 (13.0%), small vessel disease in 166 (12.5%), and cardio-embolic stroke in 226 (17.1%) patients (Table 1; Table S3 for cardio-embolic causes). Other determined cause of stroke was present in 287 (21.7%) patients (Table 2) and multiple causes in 79 (6.0%) patients (Table S4). In 333 (25.2%) patients, the stroke had an undetermined cause and was classified as cryptogenic. Patients with a cryptogenic stroke were younger (median age of 43.3 years; IQR [36.2–46.7]), than those with noncryptogenic stroke (median age 44.6; IQR [39.1–47.6]; *P*=0.001).

**Table 2.**
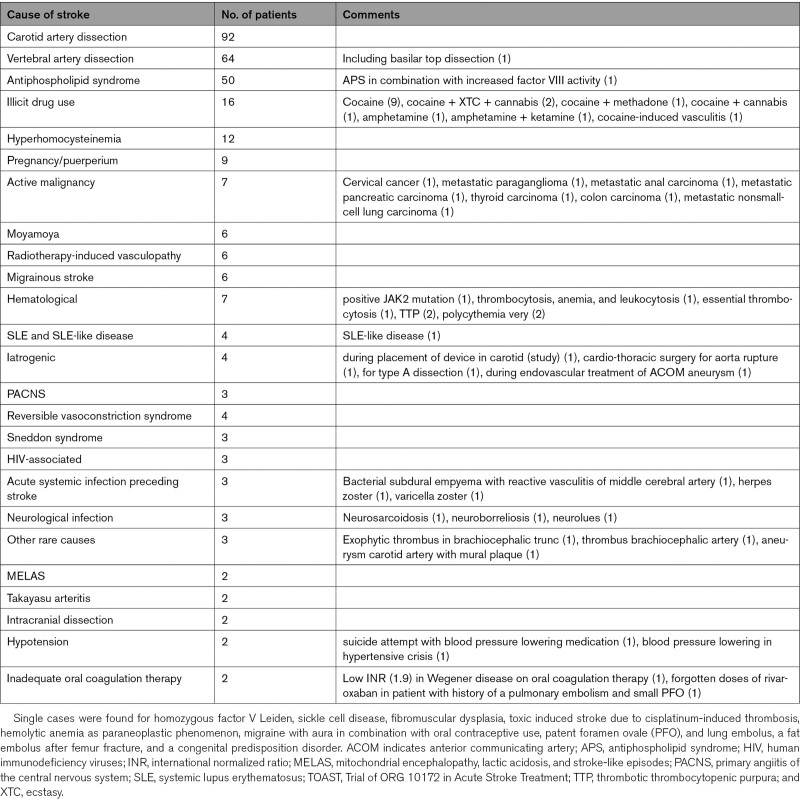
Other Determined Causes of Stroke (Within TOAST Other Determined and Multiple Causes Category)

The cause of stroke according to the TOAST classification differed between age-groups (Figure; Table S5).

**Figure.**
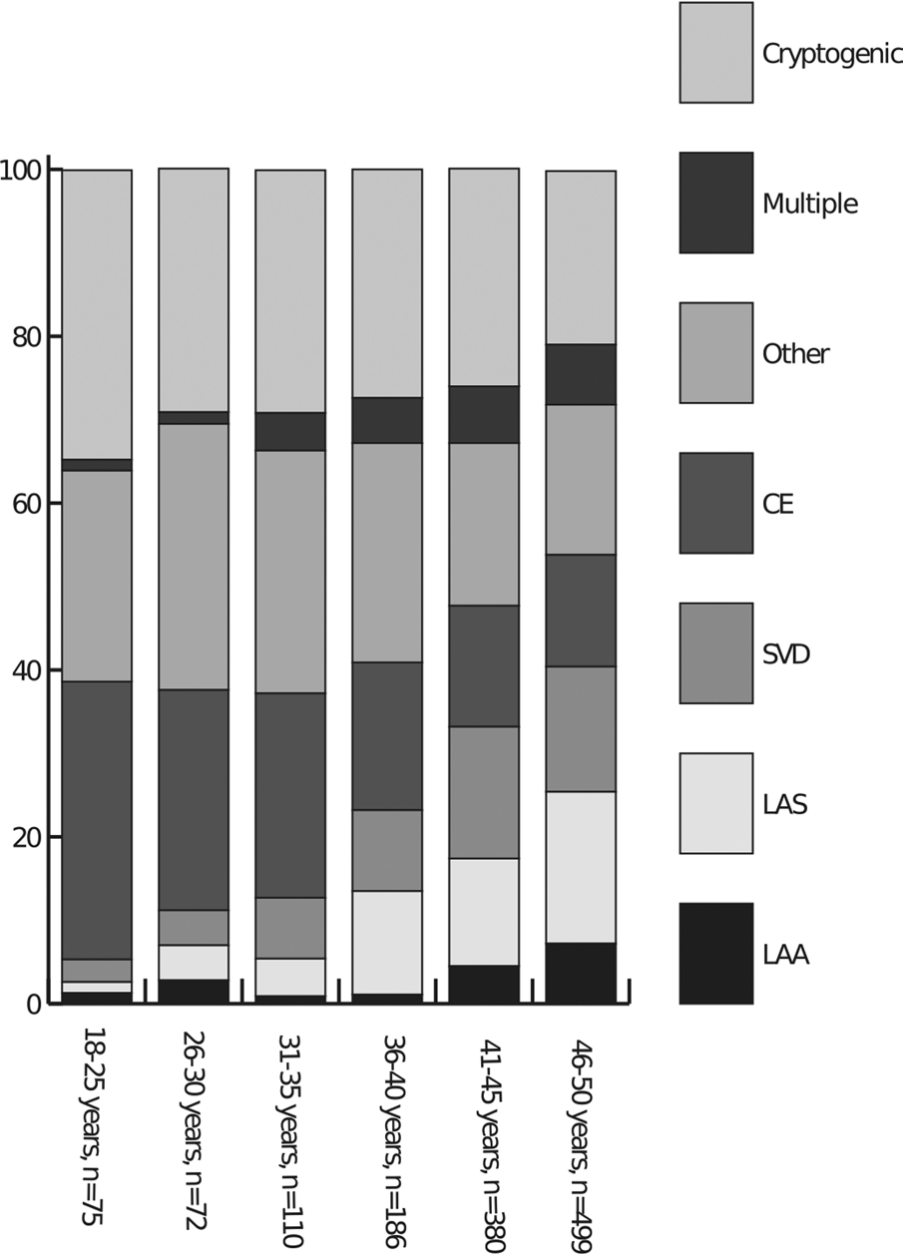
**TOAST causes distribution among different age-groups.** Significant differences between age groups and the percentages of the smallest groups that are not shown in the Figure are shown in Table S5. CE indicates cardioembolic stroke; LAA, large artery atherosclerosis; LAS, likely atherothrombotic stroke; Other, other determined cause of stroke; and SVD, small vessel disease.

### ASCOD Classification

Of the 333 patients with a cryptogenic stroke according to the TOAST classification, 73 could be assigned to 1 of the ASCOD categories (Table S6). The 260 patients in whom the stroke remained cryptogenic were younger (median age 43.1 years; IQR [36.4–46.5]) than the 1062 patients classified otherwise according to TOAST and ASCOD (median age 44.6; IQR [39.0–47.6]; *P*<0.001).

### IPSS Classification

Distribution of risk factors according to the IPSS is shown in Table 3 for the whole cohort and for patients with a cryptogenic stroke according to the TOAST criteria. Of the 333 patients, 18 (5.4%) reported illicit drug use in the year before stroke. Coagulation abnormalities were found in 85 of 333 patients (25.5%). Given that the risk factors migraine, oral contraceptive use and smoking in the IPSS are known for their increased risk of stroke in interaction,^16,17^ these were investigated: Migraine was present in 92 of the 333 patients (27.6%), of whom 34 women (18.9%) also used oral contraceptives. An estimated 122 of the 333 patients with a cryptogenic stroke according to the TOAST criteria (36.6%) were smokers, of whom 31 women (17.3%) used oral contraceptives. Thirteen women (7.3%) used both oral contraceptives, had migraine and were smokers.

**Table 3.**
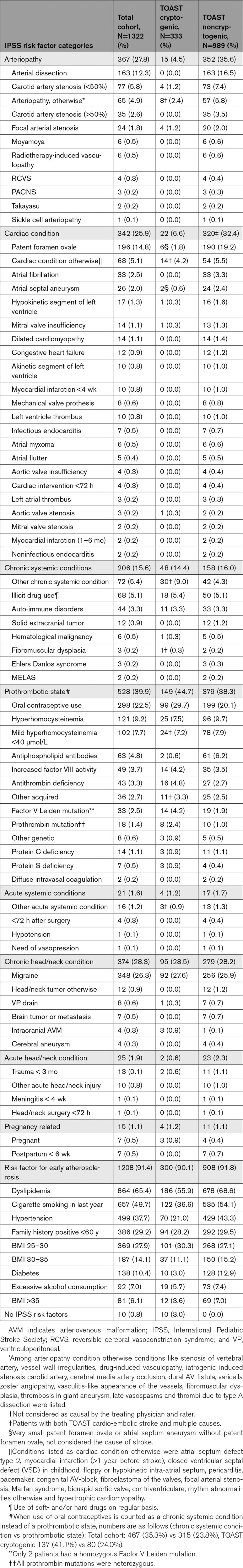
IPSS Risk Factor Categories for All Patients, Cryptogenic Patients According to TOAST and All Noncryptogenic Patients According to TOAST Criteria

In 10 of the 333 patients with cryptogenic stroke, according to the TOAST criteria (3.0%), not a single IPSS risk factor could be identified (Table 3). The group of 10 patients without any IPSS risk factor consisted of 5 men and 5 women (median age 42.3 years; 36.6–45.8). Eight of these patients had an ischemic stroke with symptoms lasting >24 hours, and 2 had symptoms lasting <24 hours.

### Differences Between the IPSS Classification in Cryptogenic and Noncryptogenic Strokes According to TOAST Classification

In comparison with patients with a noncryptogenic stroke according to TOAST, patients with a cryptogenic stroke less often had arteriopathies (4.5% versus 35.6%) and cardiac conditions (6.6% versus 32.4%), more often had a prothrombotic state (44.7% versus 38.3%) and a chronic systemic condition (9.0% versus 4.3%; Table 3). Risk factors for early atherosclerosis were highly prevalent in both groups (90.1% versus 91.8%).

## Discussion

We found that 25.2% of young patients with an ischemic stroke had a cryptogenic stroke according to the modified TOAST and 19.7% when further classified according to ASCOD criteria. Only 0.8% of 1322 patients had a cryptogenic stroke without any potential risk factors when applying the IPSS classification.

Using the TOAST classification, we found a lower percentage of patients with a cryptogenic stroke than most European cohort studies.^6,18^ This might be due to both our systematic approach and to differences in time period between studies, since radiological, cardiac and laboratory testing have become more extensive over the last decade resulting in more identifiable causes. Both the TOAST and ASCOD classification, though often applied in young patients with stroke, were not designed to classify stroke in the young. This might explain why 46.9% of all strokes are classified as other determined- and cryptogenic strokes. Although the ASCOD classification separates patients with an arterial dissection from the other determined causes, other categories are similar to the TOAST classification. As such ASCOD has limited added value in clinical practice to identify additional causes of stroke. Current classification systems developed for stroke patients in general (often >65 years) seem therefore inadequate to classify stroke causes and risk factors in young adults.

Evaluating patients with the IPSS classification can broaden the view of clinicians on potential risk factors, or factors that in interaction can play a role, such as oral contraceptive use, migraine, and smoking.^16,17^ The IPSS may shed light on (new) mechanisms that potentially lead to stroke in young adults, by subdividing risk factors in many different categories, based on a presumed pathophysiological mechanism. These different categories may help by forming a foundation for constructing a classification more specific for young adults with stroke. In contrast, the TOAST and ASCOD only have separate categories for large and small vessel disease, cardio-embolism, and dissection (ASCOD).

Although we found a potential risk factor in many patients according to the IPSS classification, this is not synonymous with causality. Classical concepts about the cause of a disease include an event, condition, or characteristic that plays an essential role in producing an occurrence of the disease, and multiple component causes can act in combinations to produce different sufficient causes for a disease.^19^ In addition, strength of association, consistency, specificity, temporality, biological plausibility of a risk factor, and the occurrence of stroke support causality.^19^ For many of the risk factors included in the IPSS, causality remains to be demonstrated. However, this also holds true for some of the causes in the TOAST and ASCOD systems (eg, mild hyperhomocysteinemia). Searching for risk factors in the large group of young adults with cryptogenic stroke using a risk factor system designed for children and adolescents can provide leads for new causes and potential treatable targets, but also help in developing a more structured, cost-effective diagnostic work-up linked to an etiological classification. Evaluating causality of certain common yet presumed risk factors in young adults with stroke would be an important area of future research,^20^ and to further prove causality, evaluation of the long-term prognosis for patients with different presumed risk factors is key, as is comparison of the prevalence of some presumed risk factors in the general population. Examples we found in this study are the high number of young adults with coagulation abnormalities with or without a patent foramen ovale^21^ in both the cryptogenic and noncryptogenic category. Also, cannabis is frequently used among young adults, often with concomitant use of alcohol and other substances, but it is rarely considered the cause of stroke. Meanwhile, a study in the United States showed a rising trend in hospital admissions for cardiovascular events in young adults due to cannabis use without concomitant use of other substances.^22^ However, studies on differences in the prevalence of these coagulation abnormalities between patients and the healthy population, as well as studies on the long-term effects and causality of cannabis on cardiovascular outcomes are scarce.^23^

Our study has several strengths. First, this study is a large, prospective cohort study investigating stroke in young adults in the last decade. Patients were therefore analyzed and treated according to the latest guidelines including magnetic resonance imaging an extensive cardiac work-up. The large number of patients allowed for detailed subgroup analysis between men and women, different age-groups, stroke subtype and different etiologies. Second, stroke misclassification was not present as all strokes were confirmed on neuroimaging, and checked independent from the treating physicians. Third, information was gathered in both a detailed and structured manner and verified by 2 from a pool of 4 investigators. Risk factor and cause designation was done systematically by 4 raters, checked by one another afterwards in 100 cases and overall discussed to reduce the interrater variability. Fourth, the study has a nation-wide character, with consecutive patients from both academic and nonacademic hospitals in different regions of the Netherlands. By also including patients who visited outpatient clinics (minor strokes) and patients who died within the first 30-days of stroke (very severe) selection bias was limited. In addition, this provides a good reflection of the stroke population of young adults and increases the generalizability of our results to other European countries.

## Limitations

Our study also has limitations. First, there were no demands on diagnostic work-up for hospitals that participated in the ODYSSEY study, and therefore, the 333 patients classified as cryptogenic according to TOAST included some incomplete work-up cases. For example, 8.1% of our patients without a cause of stroke did not receive a cardiac ultrasound, or had a transthoracic echocardiography without bubble study, potentially leading to overestimation of the number of cryptogenic strokes and an underestimation of the number of patent foramen ovale. Similarly, intracranial vascular imaging by computed tomography angiography or magnetic resonance angiography was not performed in 24% of our patients and not all cryptogenic patients received complete laboratory testing for antiphosholipid syndrome. However, we think that, for example, a missed diagnosis of vasculitis seems highly unlikely in patients with unavailable intracranial vascular imaging without anamnesis of headache, without other inflammatory laboratory parameters and with neuro-imaging without strokes in multiple territories. Regarding patients without complete laboratory testing for antiphosholipid syndrome, stroke was the first thrombo-embolic event for all patients, and the women (44%) had no history of pregnancy complications. Second, all 17 participating centers included consecutive patients, although this was not always possible, for example, during the COVID period (March 2020–February 2021). However, these were most likely missing at-random. Third, besides the standardized questionnaires, all patient’s files were checked for all the risk factors in the IPSS; however, on some risk factors such as illicit drug use, amount of alcohol consumption, and cigarette smoking, patient’s answers could possibly have not been honest in the questionnaire and in medical history taking with their treating physician, leading to an underestimation of some of these risk factors. In contrast, attribution bias might have played a role, since all patients with stroke have been seen by a neurologist, and the professional tendency to attribute stroke to a cause. Therefore, over reporting of risk factors by patients might have occurred.

## Conclusions

Current stroke classification systems developed for stroke in general (often in patients over 65 years) cannot identify the cause of stroke in many patients, leaving a large group of cryptogenic strokes in young adults <50 years of age. Many additional potential risk factors for stroke can be found using a risk factor classification system designed for children and adolescents, providing leads for (new) causes, although further research should elaborate on the actual causality of some of these factors and the prevalence in the general population. Whether a more extensive modified pediatric risk factor classification can serve as a stepping stone for a new classification scheme that may aid in the development of tailored diagnostic work-up and treatment for young patients with stroke needs to be further evaluated. However, detailed, correct etiological classification is essential for future studies, both on therapy, secondary prevention and prognosis, for comparison of patients with similar diagnostic work-up and cause.

## Article Information

### Acknowledgments

Conception and design of the study, data analysis and interpretation, and drafting a significant portion of the article or figures: MSE, JIV, CJK, AMT, FEdL.

Acquisition of the data, critical revision of the article, and final approval of the version to be published: all authors.

Systematic assessment of all patient files by at least 1 of 4 raters: ME, JV, MS, or EB.

### Sources of Funding

Professor Dr. FE de Leeuw has received the Innovational Research Incentive grant (016-126-351) and the Clinical established investigator Dutch Heart Foundation grant (2014 T060). Dr. AM Tuladhar is a junior staff member of the Dutch Heart Foundation (grant number 2016T044). Professor Dr. MJH Wermer has received a VIDI grant (9171337) of the ZonMw/NOW and the Clinical established investigator Dutch Heart Foundation grant (2016T86). Professor dr. CJM Klijn reports grants of the Dutch Heart Foundation, CVON2015–01: CONTRAST, and the support of the Brain Foundation Netherlands (HA2015·01·06), all was outside the submitted work.

### Disclosures

None.

### Supplemental Material

STROBE checklist

Tables S1–S5

## Supplementary Material
