# Supplementary material for: Risk Factors and Causes of Ischemic Stroke in 1322 Young Adults
Source: Stroke. 2022 Dec 13;54(2):439–47. doi: 10.1161/STROKEAHA.122.040524 (PMC9855752; doi:10.1161/STROKEAHA.122.040524)
Supplement: Supplementary file 1 [file str-54-439-s001.pdf]

SUPPLEMENTAL MATERIAL

Supplementary Table 1. Overview of classification systems and definitions used for this study.

**TOAST classification**		
Large vessel atherosclerosis		
1a. Atherothrombotic	Atherothrombotic stroke Patients with (1) an ipsilateral internal carotid stenosis >50% (in NASCET criteria), or (2) an ipsilateral stenosis >50% of another intra/extracranial artery, or (3) mobile thrombus in the aortic arch	
1b. Likely atherothrombotic	Likely atherothrombotic stroke Patients with no evidence of atherothrombotic stroke as defined in 1a with (1) an ipsilateral internal carotid stenosis <50%, or (2) an ipsilateral stenosis <50% of another intra/extracranial artery, or (3) aortic arch plaques >4 mm in thickness without a mobile component, or (4) a history of myocardial infarction or coronary revascularization, (5) a history of documented peripheral arterial disease, or (6) at least two risk factors for atherosclerotic disease: arterial hypertension (treated or known blood pressure before stroke >140/90 mm Hg or hypertensive retinopathy), diabetes mellitus (treated or known blood fasting glucose >7 mmol/l), current smoking (or smoking stopped within the last 6 months), high cholesterol (treated or known low-density lipoprotein before the stroke >160 mg/dl or 4,1 mmol/l)	
Small vessel disease	Patients with a small deep infarct measuring <15 mm on MRI (or CT) in the territory corresponding to symptoms, in a patient presenting a clinical syndrome compatible with a small deep infarct	
Cardio-embolic	Cardiac sources are divided into high-risk and medium-risk groups based on the evidence of their relative propensities for embolism	
	**High risk sources**	**Medium risk sources**
	Mechanical prosthetic valve	Mitral valve prolapse
	Mitral stenosis with atrial fibrillation	Mitral annulus calcification
	Atrial fibrillation	Mitral stenosis without atrial fibrillation
	Left atrial/atrial appendage thrombus	Left atrial turbulence (smoke)
	Sick sinus syndrome	Atrial septal aneurysm
	Recent myocardial infarction (< 4 weeks)	Patent foramen ovale
	Left ventricular thrombus	Atrial flutter
	Dilated cardiomyopathy	Lone atrial fibrillation
	Akinetic left ventricular segment	Bioprosthetic cardiac valve
	Atrial myxoma	Nonbacterial thrombotic endocarditis
	Infective endocarditis	Congestive heart failure
		Hypokinetic left ventricular segment
		Myocardial infarction (>4 weeks, < 6 months)
Other determined cause	Causes as shown in Table 3	
Multiple causes	Patients with two or more etiologies defined in 1–4	
Undetermined etiology / cryptogenic	Patients who did not meet criteria for the groups as defined above, maybe with incidental findings or with undetermined etiology, but incomplete evaluation.	

**ASCOD classification**	
**A: Causality grades for atherothrombosis**	
A1 (potentially causal)	Atherothrombotic stroke defined as:<br>(1) ipsilateral atherosclerotic stenosis between 50 and 99% in an intra- or extracranial artery supplying the ischemic field; or<br>(2) ipsilateral atherosclerotic stenosis <50% in an intra- or extracranial artery with an endoluminal thrombus supplying the<br>ischemic field; or<br>(3) mobile thrombus in the aortic arch; or<br>(4) ipsilateral arterial occlusion in an intra- or extracranial artery with evidence of underlying atherosclerotic plaque<br>supplying the ischemic field
A2 (causal link is uncertain)	(1) ipsilateral atherosclerotic stenosis 30–50% in an intra- or extracranial artery supplying the ischemic field; or<br>(2) aortic plaque ≥4 mm without mobile lesion

A3 (causal link is unlikely, but disease is present)	(1) plaque (stenosis <30%) in an intra- or extracranial artery, ipsilateral to the infarct area;<br>(2) aortic plaque <4 mm without mobile thrombus;<br>(3) stenosis (any degree) or occlusion in a cerebral artery not supplying the infarct area (e.g. contralateral side or opposite circulation);<br>(4) history of myocardial infarction, coronary revascularization or peripheral arterial disease;<br>(5) ipsi- or bilateral atherosclerotic stenosis 50–99% with bihemispheric MR-DWI lesion
A0 (atherosclerosis not detected)	Ruling out atherosclerosis:<br>(1) extracranial arterial stenosis: one or several of the following diagnostic tests are performed and are negative: US-Duplex, CTA, MRA, XRA, or autopsy;<br>(2) intracranial arterial stenosis: one or several of the following diagnostic tests are performed and are negative: US-TCD, MRA, CTA, XRA, or autopsy;<br>(3) aortic arch atheroma: TEE with specific assessment of the aortic arch (when the probe is pulled back at the end of the cardiac examination, turn the probe counter clockwise and take time to watch the aortic arch) or specific aortic arch assessment with CTA
A9 (incomplete workup)	US-Duplex, US-TCD or CTA, or MRA, or XRA or autopsy not performed. [A minimum workup is extra- and intracranial assessment of cerebral arteries – maximum workup also includes transesophageal assessment of the aortic arch (or a default CTA of the aortic arch)]
**S: Causality grades for small-vessel disease**	
S1 (potentially causal)	Combination of:<br>(1) lacunar infarction: small deep infarct <15 mm (in perforator branch territory) on MRI-DWI (or a default CT) in an area corresponding to the symptoms and at least one of the three following criteria:<br>(2) one or several small deep older infarct(s) of lacunar type in other territories, *and/or*<br>(3) severe (confluent – Fazekas III) leukoaraiosis, or microbleeds, or severe dilatation of perivascular spaces ('état criblé');<br>(4) repeated, recent (<1 month), TIAs attributable to the same territory as the index infarct
S2 (causal link is uncertain)	(1) only one, recent, lacunar infarction and no other abnormality on MRI (or CT) *or*<br>(2) clinical syndrome suggestive of a deep branch artery stroke, without ischemic lesion in the appropriate area seen on MRI or CT<br>(main clinical syndrome suggesting a deep branch artery – lacunar – stroke: pure hemiparesis, pure hemisensory loss, ataxic hemiparesis, dysarthria-clumsy hand syndrome, unilateral sensorimotor deficit, others: hemichorea, hemiballism, pure dysarthria, etc.)
S3 (causal link is unlikely, but disease is present)	Severe (confluent – Fazekas III) leukoaraiosis visible on MRI and/or CT scan, and/or microbleeds visible on T2*-weighted MRI, and/or severe dilatation of perivascular spaces (visible on T2-weighted MRI), and/or one or several old, small deep infarcts of lacunar type
S0 (small vessel disease not detected)	Ruling out small-vessel disease stroke: negative MRI (T2, FLAIR, GRE, DWI) and no appropriate clinical syndrome suggestive of a deep branch artery stroke
S9 (incomplete workup)	MRI (or CT) not performed
**C: Causality grades for cardiac pathology**	
C1 (potentially causal)	Cardiogenic stroke defined as acute, or recent and older bihemispheric or supra- and infratentorial territorial or cortical ischemic lesions and signs of systemic embolism with detection of at least one of the following potential causes:<br>(1) mitral stenosis (surface <1.5 cm 2);<br>(2) mechanical valve;

	(3) myocardial infarction within 4 weeks preceding the cerebral infarction;<br>(4) mural thrombus in the left cavities;<br>(5) aneurysm of the left ventricle;<br>(6) history or presence of documented atrial fibrillation – whether paroxysmal (>60 s), persistent or permanent – or flutter,<br>with or without left atrial thrombus or spontaneous echo;<br>(7) atrial disease (tachycardia-bradycardia syndrome);<br>(8) dilated or hypertrophic cardiomyopathies;<br>(9) left ventricle ejection fraction <35%;<br>(10) endocarditis;<br>(11) intracardiac mass;<br>(12) PFO *and* thrombus in situ;<br>(13) PFO *and* concomitant pulmonary embolism or proximal DVT preceding the index cerebral infarction;
C2 (causal link is uncertain)	Regardless of stroke pattern:<br>(1) PFO + atrial septal aneurysm;<br>(2) PFO and pulmonary embolism or proximal DTV concomitant but NOT preceding the index cerebral infarction;<br>(3) intracardiac spontaneous echo-contrast;<br>(4) apical akinesia of the left ventricle and decreased ejection fraction (but >35%);<br>(5) history of myocardial infarction or palpitation and multiple brain infarction, repeated either bilateral or in two different<br>arterial territories (e.g. both anterior and posterior circulation);<br>(6) no direct cardiac source identified, but multiple brain infarction, repeated either bilateral or in two different arterial<br>territories (e.g. both anterior and posterior circulation) and/or evidence of systemic emboli: renal or splenic or mesenteric<br>infarction (on CT, MRI or autopsy) or embolism in peripheral artery supplying arm or leg
C3 (causal link is unlikely, but the disease is present)	One of the following abnormalities present in isolation: PFO, ASA, strands, mitral annulus calcification, calcification aortic<br>valve, nonapical akinesia of the left ventricle, transient atrial fibrillation <60 s, atrial hyperexcitability
C0 (cardiac pathology not detected or not suspected)	Ruling out a cardiac source of embolism: minimum is negative ECG and examination by a cardiologist; maximum is negative<br>ECG/telemetry/24-hour Holter ECG/long-term ECG recording (implantable device, transtelephonic ECG, loop recorder) and<br>negative TEE for atrium, valves and septal abnormalities, negative TTE for PFO and assessment of left ventricle, negative<br>cardiac CT/MRI, negative abdominal CT/MRI (search for old or simultaneous subdiaphragmatic visceral infarction)
C9 (incomplete workup)	Minimum is ECG and examination by a trained cardiologist in the absence of cardiac imaging
**O: Causality grades for other causes**	
O1 (potentially causal)	(1) dolichoectasia with complicated aneurysm;<br>(2) polycythemia vera or thrombocythemia >800,000/mm 3;<br>(3) systemic lupus;<br>(4) disseminated intravascular coagulation;<br>(5) antiphospholipid antibody syndrome (including >100 GPL units or lupus anticoagulant);<br>(6) Fabry's disease;<br>(7) coexisting meningitis;<br>(8) sickle cell disease;<br>(9) ruptured intracranial aneurysm with or without vasospasm of the artery supplying the infarcted area;<br>(10) severe hyperhomocysteinemia;<br>(11) Horton's disease;<br>(12) other cerebral inflammatory or infectious angiitis;<br>(13) moyamoya disease, etc.
O2 (causal link is uncertain)	(1) saccular aneurysm (with a suspicion of embolism from it)<br>(2) coincidental migraine attack with neurological deficit lasting >60 min in patients with history of migraine aura
O3 (causal link is	(1) arteriovenous malformation;

unlikely but the disease is present)	(2) thrombocythosis <800,000/mm 3 ;<br>(3) antiphospholipid antibody <100 GPL units;<br>(4) homocysteinemia <40 µmol/l;<br>(5) malignoma with associated hypercoagulation (high D-dimer levels), deep vein thrombosis or pulmonary embolism and/or recent chemotherapy
O0 (no other cause detected)	Ruling out other causes: negative: cerebrospinal fluid, complete hemostasis, cerebral arterial imaging, family history of inherited disease, inflammatory markers (erythrocyte sedimentation rate, C-reactive protein), hematologic tests (platelet, leucocytes, and eosinophilic counts, hematocrit), specific tests according to the suspected disease (e.g. genetic test, retinal angiography for Susac syndrome)
O9 (incomplete workup)	Unable to reasonably exclude other causes based on best available diagnostic tests and stroke-specific history
**D: Causality grades for dissection**	
D1 (potentially causal)	(1) arterial dissection by direct demonstration (evidence of mural hematoma: hypersignal on FAT-saturated MRI or at autopsy or on TOF-MRA or CT on axial sections showing both enlargement of the arterial wall by the hematoma with narrowing of the lumen or on echography showing an hypoechoic arterial wall with narrowing of the lumen and sudden enlargement of the carotid or vertebral (V2) artery diameter;<br>(2) arterial dissection by indirect demonstration or by less sensitive or less specific diagnostic test (only long arterial stenosis beyond the carotid bifurcation or in V2, V3 or V4 without demonstration of arterial wall hematoma: on X-ray angiography, and/or echography and/or CTA and/or MRA) or unequivocal US with recanalization during follow-up
D2 (causal link is uncertain)	(1) arterial dissection by weak evidence (suggestive clinical history, e.g., painful Horner's syndrome or past history of arterial dissection);<br>(2) imaging evidence of fibromuscular dysplasia of a cerebral artery supplying the ischemic field
D3 (causal link is unlikely but disease is present)	(1) kinking or dolichoectasia without complicated aneurysm or plicature;<br>(2) fibromuscular dysplasia on arteries not supplying the ischemic field
D0 (no dissection detected or suspected)	Ruling out dissection: negative FAT-saturated MRI of suspected artery or good quality, normal X-ray angiography (too early FAT-saturated MRI performed within 3 days of symptom onset can be falsely negative and then should be repeated). If there is no clinical suspicion of dissection, the patient can be classified D0 provided good-quality extra- or intracranial cerebral artery and cardiac evaluations have been performed
D9 (incomplete workup)	In patients aged less than 60 years and with no evidence of A1, A2, S1, C1, or O1 category: no FAT-saturated MRI performed on the extra- or intracranial artery supplying the ischemic field or no X-ray angiography performed (all performed within 15 days of symptom onset)

Modified IPSS classification

Arteriopathy	Focal cerebral arteriopathy<br>Moyamoya<br>Arterial dissection<br>CADASIL<br>Radiotherapy induced vasculopathy<br>Reversible vasoconstriction syndrome<br>Giant cell arteritis<br>Takayasu arteritis<br>Primairy angiitis of the nervous system<br>Significant carotid stenosis (>50%)<br>Non-significant carotid stenosis (<50%)<br>Vasculitis

	Sickle cell arteriopathy
	Post varicella arteriopathy
	Other
	Unspecified arteriopathy
Cardiac disorders	Congenital heart disease
	Acquired heart disease
	Mechanical valve prothesis
	Mitral valve stenosis
	Mitral valve insufficiency
	Atrial fibrillation
	Sick sinus syndrome
	Recent myocardial infarction (< 4 weeks)
	Myocardial infarct (>4 weeks-6months)
	Isolated PFO
	Atrial septal aneurysm
	Dilated cardiomyopathy
	Thrombus in left ventricle
	Thrombus in left atrium
	Akinetic segment left ventricle
	Hypokinetic segment left ventricle
	Biological valve prothesis
	<72 hours after cardiac surgery
	Previous cardiac surgery
	Arrhythmia otherwise
	Infectious endocarditis
	Non-bacterial endocarditis
	Aortic valve stenosis
	Aortic valve insufficiency
	Other
Chronic systemic condition	Fabry's disease
	Fibromuscular dysplasia
	Ehlers Danlos
	Sickle cell disease
	Genetic condition (except CADASIL and genetic coagulation disorders)
	Auto-immune condition
	Hematological malignancy
	Solid extracranial tumor
	MELAS
	Illicit drug use (soft- and/or hard drugs or regular base)
	Other
Prothrombotic state	Factor V Leiden
	Prothrombin mutation
	Protein S deficiency
	Protein C deficiency
	Antithrombin deficiency
	Increased factor VIII activity
	Hyperhomocysteinemia
	Antiphospholipid syndrome
	Diffuse intravasal coagulation
	Use of oral contraceptives
	Other genetic coagulation disorder
	Other acquired coagulation disorder (HIT, TTP)
Acute systemic condition	<72 hours after surgery
	Hypotension at time of event (<90mmHg systole/60 diastole)
	Sepsis (according to SOFA criteria)
	Shock (need of vasopressive agents for a MAP >65 and serum lactate >2 mmol/l)
	Other
Chronic head/neck condition	Migraine
	Brain tumor or metastasis
	VP drain
	Cerebral aneurysm
	Intracranial AVM

	Other head/neck tumor<br>Other head/neck condition
Acute head/neck condition	Trauma head/neck <3 months<br>Tonsillar abces < 4 weeks<br>Meningitis < 4 weeks<br>Head/neck surgery < 72 hours<br>Other
Pregnancy related	During pregnancy<br>During puerperium (< 6 weeks after delivery)
Risk factors for early atherosclerosis	Hypertension (systolic >140 and/or diastolic >90 24hrs after event)<br>Smoking (at least 1 cigarette in the past year)<br>Alcohol misuse (>200g of alcohol/week =20 units)<br>Dyslipidemia (total cholesterol >5.0 mmol/l and/or LDL>3.0 and/or HDL<1.0)<br>Diabetes mellitus (sober glucose > 7.0 twice or Hba1c > 48mmol/l)<br>BMI > 25<br>Family history positive (1 degree family member with cardiovascular disease < 60 years)

Supplementary Table 2A-C. Proportion of patients with A) neurovascular imaging, B) cardiac rhythm investigations and C) cardiac ultrasound investigation, by modality.

**A***	**Neurovascular imaging in total**	**CTA**	**MRA**	**Carotid ultrasound**	**DSA**
**Patients (%)**	1269 (96%**)	658 (49.8%)	626 (47.4%)	517 (39.1%)	9 (0.7%)
**B***	**Cardiac monitoring in total**	**ECG**	**Prolonged monitoring (>24, <168 hours)**	**Prolonged monitoring (7 days)**	
**Patients (%)**	1279 (97%)	1279 (96.7%)	1011 (76.5%)	120 (9.1%)	
**C***	**Cardiac ultrasound in total**	**TTE**	**TEE**	**2nd TTE**	
**Patients (%)**	1106 (83.7%)	1071 (81.0%)	249 (18.9%)	37 (2.8%)	

* Categories are not mutually exclusive.

** In the 4% without neurovascular imaging this was deemed unnecessary due to posterior circulation stroke without suspicion of vertebral artery dissection.

Supplementary Table 3. Sources of cardio-embolism in patients with a cardio-embolic stroke.

	**Number of patients**
**High risk sources of cardio-embolism**	
Atrial fibrillation	25
Multiple high-risk sources	17
Dilated cardiomyopathy	11
Myxoma	6
Mechanic valve prothesis	5
Infectious endocarditis	5
Myocardial infarction < 4 weeks	4
Left ventricle thrombus	4
Left atrial thrombus	2
Akinetic segment of left ventricle	2
Mitral valve stenosis with AF	2
Sick sinus syndrome	1
**Medium risk sources of cardio-embolism**	
Patent foramen ovale alone	158
Multiple medium risk sources	24*
Hypokinetic segment of left ventricle	7
Mitral valve insufficiency	6
Congestive heart failure	5
Atrial septum aneurysm	3
Non-infectious endocarditis	2
Atrial flutter	2

AF: atrial fibrillation, PFO: patent foramen ovale

*: 18 patients had a PFO in combination with an atrial septum aneurysm

Supplementary Table 4. Distribution of 79 patients with multiple causes among TOAST categories.

	LAA	LAS	SVD	CE	Other	Unknown
**LAA**	x	0	0	2	5	0
**LAS**	0	x	2	20	17	0
**SVD**	0	2	x	5	6	0
**CE**	2	20	5	x	16	0
**Other**	5	17	6	16	x	4
**Unknown**	0	0	0	0	4	x

LAA: Large artery atherosclerosis, LAS: likely atherothrombotic stroke, SVD: small vessel disease, CE: cardioembolic stroke, Other: Other determined cause of stroke

Supplementary Table 5. TOAST etiology distribution among different age-groups.

	LAA	LAS	SVD	CE	Other	Multiple	Cryptogenic
**18-49 years median age (IQR)**	46.6 years (4.2)	46.3 years (5.9)	45.5 years (5.3)	42.3 years (12.7)	42.2 years (11.4)	45.3 years (6.9)	43.3 years (10.4)
**18-25 years** (n=75, (%))	1 (1.3)	1 (1.3)	2 (2.7)	25 (33.3)	19 (25.3)	1 (1.3)	26 (34.7)
**26-30 years** (n=72, (%))	2 (2.8)	3 (4.2)	3 (4.2)	19 (26.4)	23 (31.9)	1 (1.4)	21 (29.2)
**31-35 years** (n=110, (%))	1 (0.9)	5 (4.5)[a]	8 (7.3)	27 (24.5)	32 (29.1)	5 (4.5)	32 (29.1)
**36-40 years** (n=186, (%))	2 (1.1)	23 (12.4)[a,c]	18 (9.7)[a]	33 (17.7)[a]	49 (26.3)	10 (5.4)	51 (27.4)
**41-45 years** (n=380, (%))	17 (4.5)[c]	49 (12.9)[a,b]	60 (15.8)[a,b,c]	55 (14.5)[a,b,c]	74 (19.5)[b,c]	26 (6.8)	99 (26.1)
**46-49 years** (n=499, (%))	36 (7.2)[c,d]	91 (18.2)[b,c,d]	75 (15.0)[b,c]	67 (13.4)[b,c]	90 (18.0)[b,c,d]	36 (7.2)	104 (20.8)[a]

LAA: Large artery atherosclerosis, LAS: likely atherothrombotic stroke, SVD: small vessel disease, CE: cardioembolic stroke, Other: Other determined cause of stroke

a: Significant compared to age group 18-25

b: Significant compared to age group 26-30

c: Significant compared to age group 31-35

d: Significant compared to age group 36-40

e: Significant compared to age group 41-45

Supplementary Table 6. Distribution of patients along ASCOD categories. (A) All 1322 patients and (B) 73 of 333 patients with cryptogenic stroke according to TOAST that fall into one of the ASCOD categories.

A	N (%)		N (%)		N (%)		N (%)		N (%)
**A0**	883 (66.8)	**S0**	925 (70.0)	**C0**	797 (60.3)	**O0**	851 (64.4)	**D0**	847 (64.1)
**A1**	72 (5.4)	**S1**	87 (6.6)	**C1**	97 (7.3)	**O1**	127 (9.6)	**D1**	165 (12.5)
**A2**	31 (2.3)	**S2**	92 (7.0)	**C2**	33 (2.5)	**O2**	21 (1.6)	**D2**	3 (0.2)
**A3**	114 (8.6)	**S3**	59 (4.5)	**C3**	183 (13.8)	**O3**	135 (10.2)	**D3**	1 (0.1)
**A9**	222 (16.8)	**S9**	159 (12.0)	**C9**	212 (16.0)	**O9**	188 (14.2)	**D9**	306 (231)
**Total**	1322 (100)	**Total**	1322 (100)	**Total**	1322 (100)	**Total**	1322 (100)	**Total**	1322 (100)
B	N (%)		N (%)		N (%)		N (%)		N (%)
**A0**	47 (64.4)	**S0**	50 (68.5)	**C0**	56 (76.5)	**O0**	30 (41.1)	**D0**	56 (76.7)
**A1**	1 (1.4)	**S1**	0 (0.0)	**C1**	0 (0.0)	**O1**	1 (1.4)	**D1**	0 (0.0)
**A2**	3 (4.1)	**S2**	2 (2.7)	**C2**	1 (1.4)	**O2**	3 (4.1)	**D2**	1 (1.4)
**A3**	12 (16.4)	**S3**	13 (17.8)	**C3**	8 (11.0)	**O3**	35 (47.9)	**D3**	1 (1.4)
**A9**	10 (13.7)	**S9**	7 (9.6)	**C9**	8 (11.0)	**O9**	4 (5.5)	**D9**	15 (20.5)
**Total**	73 (100)	**Total**	73 (100)	**Total**	73 (100)	**Total**	73 (100)	**Total**	73 (100)

Table A (ASCOD classification for all 1322 patients):
Of all 1322 patients, 194 patients had a zero for all categories, which means after evaluation no cause was identified.
Of the patients with a 9 (incomplete work-up) in one or more of the categories, 163 patients had no other possible cause (A-S-C-O-D with 1, 2 or 3) in one of the other categories. Only 2 patients had an incomplete work-up (9) for all five subcategories.

Table B (ASCOD classification for 73 cryptogenic patients according to TOAST, the other 260 were also cryptogenic according to the ASCOD classification):
The causal link was believed to be very uncertain in the patients with A1 and O1, who were therefore classified as having a cryptogenic stroke according to the TOAST classification by 4 raters.
